# Recurrent Interpopulation Selection in Popcorn: From Heterosis to Genetic Gains

**DOI:** 10.3390/plants12051056

**Published:** 2023-02-27

**Authors:** Divino Rosa dos Santos Junior, Antônio Teixeira do Amaral Junior, Valter Jário de Lima, Jhean Torres Leite, Rosimeire Barboza Bispo, Valdinei Cruz Azeredo, Janeo Eustáquio de Almeida Filho, Samuel Henrique Kamphorst, Flávia Nicácio Viana, Rodrigo Moreira Ribeiro, Alexandre Pio Viana, Geraldo de Amaral Gravina

**Affiliations:** Laboratório de Melhoramento Genético Vegetal, Centro de Ciências e Tecnologias Agropecuárias (CCTA), Universidade Estadual do Norte Fluminense Darcy Ribeiro—UENF, Campos dos Goytacazes 28013-602, Brazil

**Keywords:** popping expansion, genetic gains, genetic parameters

## Abstract

In view of the need to develop new popcorn cultivars and considering the uncertainties in choosing the most appropriate breeding methods to ensure consistent genetic progress, simultaneously for both popping expansion and grain yield, this study addressed the efficiency of interpopulation recurrent selection regarding genetic gains, the study of the response in genetic parameters as well as heterotic effects on the control of the main agronomic traits of popcorn. Two populations were established, Pop_1_ and Pop_2_. A total of 324 treatments were evaluated, which consisted of 200 half-sib families (100 from Pop_1_ and 100 from Pop_2_), 100 full-sib families from the two populations and 24 controls. The field experiment was arranged in a lattice design with three replications in two environments, in the north and northwest regions of the State of Rio de Janeiro, Brazil. The genotype × environment interaction was partitioned and the genetic parameters, heterosis and predicted gains were estimated by the Mulamba and Mock index, based on selection results in both environments. The genetic parameters detected variability that can be explored in successive interpopulation recurrent selection cycles. Exploring heterosis for GY, PE and yield components is a promising option to increase grain yield and quality. The Mulamba and Mock index was efficient in predicting the genetic gains in GY and PE. Interpopulation recurrent selection proved effective to provide genetic gains for traits with predominantly additive and dominance inheritance.

## 1. Introduction

The species popcorn (*Zea mays* var. Everta L.) is native to the American continent and is used exclusively for human consumption [1]. Brazil is the second largest consumer of popcorn, while the largest market lies in the United States of America. In 2018, Brazil produced approximately 260,000 tons of popcorn, which generated a turnover of USD 628 million. Until 2024, a production increase of 48% is expected [2].

Currently, most popcorn seeds for cultivation in Brazil are imported from the USA and Argentina, which boosts production costs. The decisive characteristics for the selection of seeds are mainly good yields and high grain quality [3]. In this context, the development of popcorn cultivars with high agronomic potential, i.e., with high grain yield (GY) and high popping expansion (PE), is imperative to stimulate the expansion of popcorn cultivation with seeds selected in breeding programs in and for Brazil [4,5,6].

In regards to the genetic control and inheritance of the main agronomic traits of popcorn, since the publication of the classic study of Larish and Brewbaker (1999) [7], the control of the trait popping expansion is understood to be due exclusively to additive effect genes, and that of grain yield is primarily due to dominance effects [8,9,10,11,12]. Owing to the exclusivity of the additive effects on the expression of popping expansion, described elsewhere, the development of intrapopulation breeding methods has become a priority [8,9,13,14,15]. However, some studies have shown that dominance effects can also influence popping expansion to some extent, which indicates that the exploration of hybrids may be an equally effective option for the development of higher-yielding cultivars [16,17,18,19,20,21]. In this way, since dominance may be important for the inheritance of high PE, it is worth highlighting the use of interpopulation recurrent selection. By this method, the frequencies of favorable alleles of populations per se are gradually increased, while gains in traits with a genetic effect of dominance are potentiated by exploitation of heterosis. In addition, at the end of each cycle, this method establishes hybrids with high yield and quality potential, suited for the breeding of lines and/or for the market [22,23].

To date, only one study on interpopulation recurrent selection in popcorn has been published [24]. Thus, the following scientific questions arise: How will the variance components affect the main agronomic characteristics of popcorn when subjected to reciprocal recurrent selection? How viable is interpopulation recurrent selection in obtaining effective gains in PE in popcorn populations? How do different family structures (interpopulation of full-sib families (FSFs) versus intrapopulation of half-sib families (HSFs)) affect gains in GY and yield components and PE?

For all the above reasons, it was deemed appropriate to carry out this research with interpopulation recurrent selection in popcorn to assess possible genetic gains and investigate the behavior of genetic parameters as well as heterotic effects on the genetic control of the main agronomic traits.

## 2. Results

### 2.1. Genetic and Environmental Variability

The results of the F test in the combined analysis of variance (Table 1) indicated significant differences between genotypes for all evaluated traits. In addition, significance was detected in the partitioning of the genotype effect (group, FSF, HSF_Pop1_, HSF_Pop2_ and controls) for all traits. Although the genotype × environment (G × E) interaction caused significant phenotypic differences for all evaluated characteristics, the partitioning of the effects of this interaction showed no significance in the source of variation groups × environment for EL, as well as in the source of variation control × environment for GY, and in HSF_Pop1_ × environment for PE. On the other hand, when analyzing the type of G × E interaction, a simple type interaction was predominant for PE (54.76%); on the other hand, for GY and yield components (100 GW, EL and ED) there was a predominance of the complex type interaction (63.42%, 99.86%, 83.21% and 61.20%, respectively).

Regarding the experimental variation coefficient (CVe), only that of GY exceeded 20%, while the values of the other evaluated characteristics were lower than 10% (Table 1).

Due to the significance of the interaction (*p* ≤ 0.01) for G × E in the combined analysis, individual analysis of variance was performed for each environment. A significant treatment effect was found for all traits in both environments (Table 2). By partitioning the treatment effect, the existence of significant differences among and within groups was detected, except for the group effect for EL in Campos.

Equivalent estimates were obtained for the coefficients of environmental variation between the two environments, since at both locations, the values for GY were greater than 20% and lower than 10% for the other characteristics (Table 2).

In a comparison of the environments, overall mean estimates for GY and PE were higher in Campos (2.34 t ha^−1^ and 28.35 mL g^−1^, respectively) than in Itaocara (2.25 t ha^−1^ and 27.84 mL g^−1^, respectively). On the other hand, in the environment of Itaocara, the means for 100 GW (14.91 g), EL (16.18 cm) and ED (31.60 mm) were higher than in Campos (12.99 g, 16.17 cm and 30.66 mm, respectively).

### 2.2. Distribution of Families, Means and Heteroses

**Figure 1:**
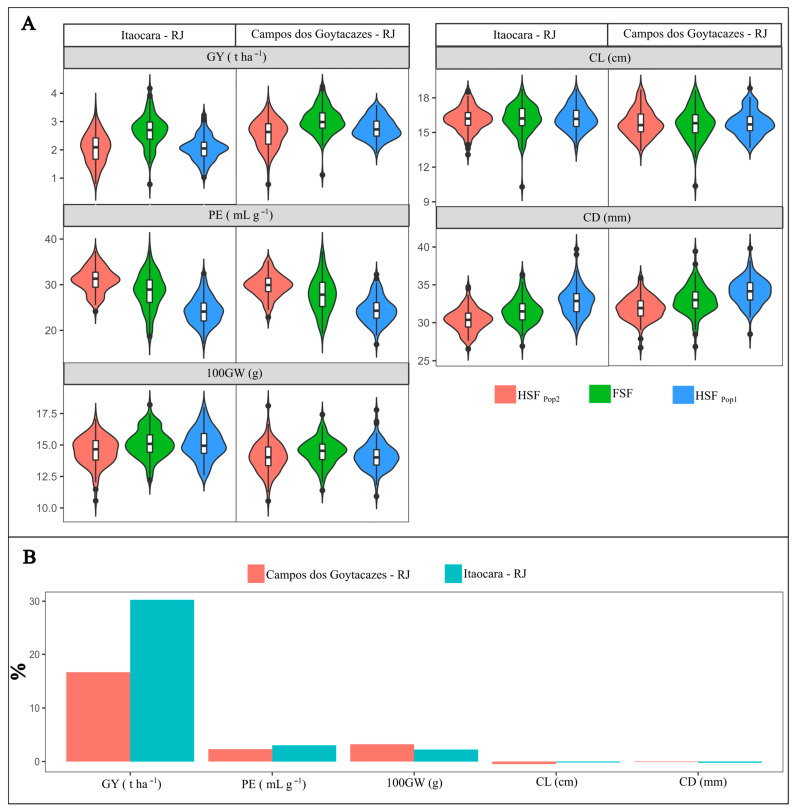
(**A**)—Box plot of distribution of family means of half-sibs and full-sibs from the popcorn populations UENF-synthetic and UENF-14 for six agronomic traits in two environments, Campos dos Goytacazes and Itaocara, in the State of Rio de Janeiro, Brazil. Within the box plots, the lateral curves represent the data distribution; horizontal lines represent the mean; lower and upper box plot boundaries represent the 25th and 75th percentiles, respectively; lower and upper whiskers represent the minimum and maximum values, respectively; and dots above and below the whiskers represent outliers. (**B**)—Estimates of heterosis for six agronomic traits of full-sib families derived from crosses between the popcorn populations UENF-synthetic and UENF-14 in environments of Campos dos Goytacazes and Itaocara. GY = mean grain yield; PE = popping expansion; 100 GW = mean 100-grain weight; EL = mean ear length; ED = mean ear diameter; FSF = full-sib family; HSF_Pop1_ = half-sib family from UENF-synthetic; and HSF_Pop2_ = half-sib family from UENF-14.

Considering the full-sib families (FSFs) and estimated heterosis values (H), the estimates varied between positive and negative. The estimated values were highest for GY (30.25% in Itaocara and 16.66% in Campos). In Itaocara and Campos, the percentage values for PE (3.02 and 2.31%) and 100 GW (2.23 and 3.23%) were low and positive. The estimates for the yield components EL and ED were negative in both environments (−0.17 and −0.21% in Itaocara and −0.47 and −0.10% in Campos, respectively; Figure 1).

Regarding yield performance, mean GY was higher for FSFs than for HSFs in both environments (2.68 t ha^−1^ in Itaocara and 2.60 t ha^−1^ in Campos). The mean grain yields of HSFs of both studied populations were equivalent in the environment of Itaocara (2.06 t ha^−1^). On the other hand, HSF_Pop1_ in Campos produced higher yields (2.32 t ha^−1^) than HSF_Pop2_ (2.13 t ha^−1^) (Figure 1).

For popping expansion, the mean PE of FSFs was close to that of HSFs (28.53 mL g^−1^ in Itaocara and 28.93 mL g^−1^ in Campos). For HSF_Pop1_, the PE estimate was 24.34 and 25.85 mL g^−1^, in Itaocara and Campos, respectively. In general, PE was highest for HSFs derived from population UENF-14, followed by HSF_Pop2_, with estimates of 31.04 mL g^−1^ in Itaocara and 28.93 mL g^−1^ in Campos.

For the mean 100-grain weight (100 GW) as well as GY, the means tended to be higher for FSFs compared to HSFs. The FSF means for the environments of Itaocara and Campos were 15.13 and 13.29 g, respectively. For HSF_Pop1_, the estimates were 15.08 and 12.9 g and for HSF_Pop2_, 14.51 and 13.83 g in Itaocara and Campos, respectively (Figure 1).

For mean ear length (EL), the FSF means were 16.17 cm for Itaocara and 16.19 cm for Campos. Considering HSF_Pop1_ for UENF-synthetic, the mean estimates were 16.25 and 16.19 cm in Itaocara and Campos, respectively. The means of HSF_Pop2_ progenies from population UENF-14 were 16.15 and 16.22 cm in Itaocara and Campos, respectively (Figure 1).

For mean ear diameter (ED) in Itaocara and Campos, the means of the tested FSFs were 31.57 and 29.73 mm; 32.88 and 31.59 mm for HSF_Pop1_; and 30.39 and 29.73 mm for HSF_Pop2_, respectively (Figure 1).

### 2.3. Genetic Components: The Key to Selection of Popcorn

For GY, the estimates of the variance components in the FSFs tested in Itaocara and Campos were as follows: genetic variance ($\sigma_{g}^{2}$) of 0.19 and 0.14; heritability ($h^{2}$) of 61.28 and 62.73%; genotypic coefficient of variation ($CV_{g})$ of 16.34 and 14.36%; and variation index ($I_{v}$) of 0.61 and 0.68%, respectively (Table 3). The variance components in the environments of Itaocara and Campos for HSF_Pop1_, were estimated as: $\sigma_{g}^{2}\;$of 0.06 and 0.05; $h^{2}$ of 34.26 and 39.35%; $CV_{g}$ of 12.21 and 9.96%; $I_{v}$ of 0.46 and 0.47%; and $\sigma_{a}^{2}$ of 0.25 and 0.21, respectively (Table 3). The following variance components were estimated for the HSF from population UENF-14 (HSF_Pop2_) in Itaocara and Campos: $\sigma_{g}^{2}\;$of 0.16 and 0.17; $h^{2}$ of 57.45 and 66.75%; $CV_{g}$ of 19.67 and 19.13%; $I_{v}$ of 0.74 and 0.90%; and $\sigma_{a}^{2}$ of 0.66 and 0.66, respectively (Table 3).

Analyzing the PE characteristic, the variance components estimated from FSFs in the environments of Itaocara and Campos were: $\sigma_{g}^{2}\;$of 13.35 and 9.90; $h^{2}$ of 84.98 and 79.75%; $CV_{g}$ of 12.81 and 10.88%; and $I_{v}$ of 1.34 and 1.12%, respectively. Considering the HSF_Pop1_ and in Itaocara and Campos, the following estimates were found: $\sigma_{g}^{2}\;$of 6.82 and 4.32; $h^{2}$ of 74.28 and 63.22%; $CV_{g}$ of 10.73 and 8.04%; $I_{v}$ of 1.12 and 0.83%; and $\sigma_{a}^{2}$ of 27.28 and 17.28, respectively (Table 3). For HSF_Pop2_ in the Itaocara and Campos environments, the following estimates were calculated: $\sigma_{g}^{2}\;$of 4.80 and 2.83; $h^{2}$ of 67.05 and 52.96%; $CV_{g}$ of 7.06 and 5.48%; $I_{v}$ of 0.74 and 0.57%; and $\sigma_{a}^{2}$ of 19.22 and 11.32, respectively (Table 3).

Evaluating the weight of 100 grains (100 GW) for FSFs in the environments of Itaocara and Campos, the variance components were estimated as: $\sigma_{g}^{2}\;$of 1.03 and 0.96; $h^{2}$ of 78.89 and 81.05%; $CV_{g}$ of 6.70 and 7.39%; and $I_{v}$ of 1.10 and 1.17%, respectively. In Itaocara and Campos, for HSF_Pop1_ the results were: $\sigma_{g}^{2}\;$of 1.20 and 1.03; $h^{2}\;$of 81.35 and 82.10%; $CV_{g}$ of 7.26 and 7.87%; $I_{v}$ of 1.19 and 1.24%; and $\sigma_{a}^{2}$ of 4.79 and 4.13. The estimates of the HSF_Pop2_ variance components, in Itaocara and Campos were: $\sigma_{g}^{2}\;$of 0.99 and 1.36; $h^{2}$ of 78.35 and 85.75%; $CV_{g}$ of 6.87 and 9.08%; $I_{v}$ of 1.13 and 1.43%; and $\sigma_{a}^{2}$ of 3.98 and 5.42, respectively (Table 3).

The estimates of the variance components for mean ear diameter (ED) of the FSF tested in the Itaocara and Campos environments were $\sigma_{g}^{2}$ of 2.55 and 2.21; $h^{2}\;$of 88.06 and 85.91%; $CV_{g}$ of 5.06 and 4.86%; and $I_{v}$ of 1.57 and 1.43%, respectively. In Itaocara and Campos for HSF_Pop1_ the estimates of variance components were $\sigma_{g}^{2}$ of 3.37 and 1.81%; $h^{2}$of 90.68 and 83.31%; $CV_{g}$ of 5.58 and 4.26%; $I_{v}$ of 1.73 and 1.25%; and $\sigma_{a}^{2}$ of 13.47 and 7.25, respectively (Table 3). Likewise, regarding the HSF_Pop2_, the estimates were $\sigma_{g}^{2}\;$of 1.94 and 1.30; $h^{2}\;$of 84.84 and 78.18%; $CV_{g}$ of 4.58 and 3.84; $I_{v}$ of 1.42 and 1.13%; and $\sigma_{a}^{2}$ of 7.75 and 5.20, respectively (Table 3).

### 2.4. Expected Selection Gains

Based on the economic weights obtained by the estimated values of $SD_{g}$, estimates of simultaneous gains in Itaocara and Campos of 1.58 and 3.48% were obtained for PE, 10.4 and 8.69% for GY; and 1.97 and 4.19% for EL, respectively. In turn, 100 GW decreased by −1.24% in Itaocara, in spite of an increase of 0.36% in Campos. For trait ED, no positive gains were obtained in either environment, but instead we observed a reduction of −0.94% for Itaocara and −0.56% for Campos (Table 4).

When $CV_{g}$ estimates were used as economic weights, the following simultaneous gains were predicted for Itaocara and Campos, respectively, for the characteristics GY, PE and EL: 0.04 and 0.21%; 2.01 and 1.79%; and 0.32 and 0.88%. The trait 100 GW was reduced by 0.06% in the environment of Itaocara and increased by 0.32% in Campos. ED decreased by 0.14 and 0.03% in Itaocara and Campos, respectively (Table 4).

From the estimated $I_{V}$ values, gains in all characteristics were estimated as: GY (4.38 and 5.14%), PE (0.46 and 1.65%), 100 GW (2.26 and 2.87%), CL (5.30 and 5.04%), and CD (2.35 and 2.21%), for Itaocara and Campos, respectively (Table 4).

From the economic weights assigned based on the $h^{2}$ estimates, simultaneous gains were expressed in the environments of Itaocara and Campos for most of the characteristics: GY (4.50 and 6.26%), 100 GW (3.01 and 2.70%), EL (4.93 and 5.09%) and ED (2.53 and 2.30%). Popping expansion decreased by −0.98% in Itaocara; however, in Campos an increase of 1.32% was predicted (Table 4).

By means of the economic weights assigned by the breeder, it was possible to obtain simultaneous and approximately equal predicted gains in both environments for the traits GY (8.72 and 8.55%), PE (8.71 and 9.68%) and EL (3.27 and 4.26%), in Itaocara and Campos, respectively. In addition, decreases in 100 GW (0.96 and 0.39%) and ED (0.39 and 1.13%), respectively, were observed (Table 4).

In Itaocara, considering the selection of the 30 best FSFs based on the economic weights assigned by the breeder for mean grain yield, 29 FSFs performed better in relation to the mean of population UENF-14, while 28 FSFs stood out over the mean of UENF-synthetic. For PE, a similar situation occurred with 13 FSFs of population UENF-14 and for the synthetic population all FSFs performed better. In a simultaneous analysis of GY and PE, 12 FSFs were superior to UENF-14 and 28 in comparison with the synthetic population (Figure S1).

In Campos, a higher mean GY was estimated for 29 FSFs in relation to that of population UENF-14, and a higher mean GY for the same number of progenies (29 FSF) in relation to the mean of the synthetic population. For mean PE, 20 FSFs performed better than population UENF-14, whereas for the synthetic population, all FSFs performed better. For both GY and PE, 12 FSFs were superior to UENF-14, while 28 stood out over the synthetic population (Figure S1).

In general, the economic weights attributed by the breeder were the most useful to reliably select the 30 best families for recombination, favoring greater simultaneous gains and partially equivalent gains for GY and PE at both evaluation locations. The increments for mean grain yield and popping expansion were 0.389 t ha^−1^ and 2.417 mL g^−1^ in Itaocara, and 0.362 t ha^−1^ and 2.621 mL g^−1^ in Campos, respectively (Figure S2).

## 3. Discussion

### 3.1. Genetic and Environmental Variability

The results of the combined analysis of variance indicate the existence of variability among and within the groups of genotypes evaluated for the studied agronomic traits, which allows the assumption of genetic gains with selection in both studied environments. However, the significance of the G × E interaction for GY, PE, 100 GW and ED can hamper the simultaneous selection of superior genotypes in the evaluated environments. In recurrent selection programs for multi-environments, the G × E interaction influences the magnitude of selection gains, the identification of homogeneous environments and definition of the most adequate selection strategy [25]. In popcorn, GY and its components are polygenic characteristics, with a strong environmental influence on trait expression [6,26,27]. Popping expansion has been claimed to be an oligogenic qualitative trait [28]. In this sense, Lu et al. (2003) [29] identified four QTLs on chromosomes 1S, 3S, 5S and 5L, which together explained 45% of the phenotypic variation in PE.

In previous cycles in the intrapopulation recurrent selection program, significant interaction effects were also observed for these two environments [5,21,30]. In this sense, although the two regions, north (Campos) and northwest (Itaocara) of the State of Rio de Janeiro [31], have similar edaphoclimatic conditions, the small differences at each location affected the phenotypic performance significantly, which confirmed the need to represent the respective regions of these two environments in the breeding program [5,21,30].

For PE, a simple G × E interaction was predominant. This type of interaction favors the selection of superior genotypes, since the relative ranking refers only to the genotype performance in different environments, unrelated to the recommendation of cultivars, since the environments are considered equal [32]. On the other hand, for GY, 100 GW, EL and ED a complex G × E interaction predominated. This finding reveals an inconsistency in the performance of some genotypes in the evaluated environments, increasing the difficulty of selecting and recommending them for both locations [32,33,34]. To mitigate this problem, some procedures can be adopted, e.g., selection of specific genotypes and a separate breeding program for each environment or the identification of genotypes with greater phenotypic stability [25,35].

In the analysis for each environment, the significant effect of genotypes, FSF, HSF_Pop1_ and HSF_Pop2_ demonstrates the existence of genetic variability among and within the evaluated populations. In relation to UENF-14, the successive selection cycles can therefore be considered successful [5,9,20,21,30,36,37,38,39,40], with genetic gains for PE and GY, without loss of genetic variability. In turn, the recombination of nine divergent lines with high general combining ability for GY and PE to constitute the UENF-synthetic population was sufficient to generate genetic variability within this population, indicating the possibility of genetic gains with selection.

According to the classification system proposed by Gomes (2009), CVe values ≤10% indicate excellent experimental precision, 10 to 20% good experimental precision, and ≥20% low experimental precision. In both environments, the CVe of PE, 100 GW, CL and CD was lower than 10%, while the experimental variations for GY exceeded 20%. According to Scapim et al. (1995) [41] and Fritsche-Neto et al. (2012) [42], the nature of the evaluated characteristics must be known to determine a more reliable pattern of experimental precision. In this context, these authors proposed a new, more robust classification criterion for maize, which associated the CVe (%) with the nature of each trait. According to this classification, GY is a trait under strong environmental influence and coefficients of variation between 20 and 30% are frequent in field experiments and are considered adequate for maize.

### 3.2. Distribution of Families, Means and Heteroses

In this study, HSF_Pop1_ and HSF_Pop2_ reflect the allelic distribution of the FSF base populations. In this context, the positive heterosis values for GY and 100 GW in the FSFs suggest higher yields than in the HSF progenies, highlighting the predominance of dominance effects of genes in the expression of these traits, due to the superiority of the mean deviation of F_1_ from the parents [25,32,43]. 

According to Falconer (1996) [43], predominance of additive gene effects in the inheritance of a trait occurs when the parental mean is equal to the mean of the F_1_ generation. In this situation, the equivalence of the mean performance of the FSFs and HSFs for PE, EL and ED shows the predominance of additive effects on the expression of these characteristics.

The predominance of additive gene action on PE and dominant for GY was observed by Larish and Brewbaker (1999) [7] in a diallel with temperate and tropical progenies; by Pereira and Amaral Júnior (2001) [8], using Comstock and Robinson’s Design I; by Freitas Júnior et al. (2006) [10], who used a mating scheme in a circulant diallel; by Rangel et al. (2011) [11], who applied the intrapopulation recurrent selection method to full-sib families; and by Cabral et al. (2016) [12], who used a complete diallel mating system.

Based on the results for PE, minor heterotic effects associated with the tendency of the mean FSF to be equivalent to the mean HSF indicates the high likeliness that additive genes control this characteristic. Thus, additive genetic variance was confirmed to be more relevant than dominance effects in the genetic control of PE [18,44]. However, dominance effects should not be disregarded, since they also influence the trait. As pointed out by Babo et al. (2006) [16] and Li et al. (2007) [17], based on crosses between popcorn and common flint corn, QTL maps were constructed that identified non-additive marks of overdominance associated with PE expression. More recently, in a study on PE inheritance, using a cross between popcorn and flint corn, Coan et al. (2019) [18] observed two genetic effects on trait inheritance: (i) predominance of additive genes together with additive and dominance polygenes (mixed inheritance); and (ii) occurrence of polygenes participating in the additive as well as dominant effects. In studies with a diallel mating scheme on PE inheritance in popcorn, Lima et al. (2019) [45], Oliveira et al. (2019) [46,47] and Santos et al. (2020) [19] stated a greater influence of dominance effects.

Among the grain yield components, 100 GW was the only trait with positive heterosis and evidence of dominance deviations. In a study on the inheritance of the main popcorn traits by means of a diallel in irrigated and water-stressed environments, Lima et al. (2019) [45] suggested the occurrence of inheritance of the dominant type for 100 GW. The positive correlation between 100-grain weight and productivity makes the former an important component of GY [48]; consequently, a reduction in the number and weight of grains affects yields directly [49,50]. In this context, exploiting heterosis is a promising genetic solution to improve 100 GW, as heterosis was one of the most decisive factors for the exponential increase in grain yield in maize in the last decades [51,52,53]. 

The heterotic effects for the traits EL and ED were negative and tended to be zero. This does not necessarily imply absence of dominance, since the sum of dominance deviations can be zero when the signs are positive and negative [43]. However, a comparison of the HSF and FSF means showed a trend closer to what is expected from an additive-type characteristic, as the FSF means approached the HSF means. On the other hand, it is worth emphasizing that dominant gene actions are commonly more important in the expression of EL and ED [45,54].

The application of breeding methods that include the exploitation of hybrids seems to be an auspicious option to raise grain yield and 100 GW [55]. However, for PE and other yield components, both additive and dominance effects ought to be taken into consideration. In this respect, reciprocal recurrent selection between populations becomes promising, as additive alleles with gains tend to be concentrated within each population and gains among populations with dominance can be explored in hybrid crosses [56].

### 3.3. Genetic Components: The Key to Selection in Popcorn

The covariance in FSFs corresponds to 0.5 of the additive variance $\left(\sigma_{a}^{2}\right)$ added to 0.25 of the dominance variance $\left(\sigma_{d}^{2}\right)$, whereas covariance in HSFs corresponds to 0.25 of $\sigma_{a}^{2}$ [43]. Apparently, the genetic variances ($\sigma_{g}^{2}$) for all traits were higher for FSFs than HSFs or close to the highest $\sigma_{g}^{2}$ of HSFs, except for 100 GW in Campos. These results may be evidence of the presence of dominance effects in the FSFs, even though no direct relationship between the $\sigma_{g}^{2}$ estimates between the two population structures could be observed, since the plants that gave rise to the HSFs were not the same as those crossed to obtain the plants of the FSFs.

The heritability coefficient expresses the reliability of the phenotypic value as a guide to the genotypic value [25,43]. Lower heritability values for GY (34–67%) compared to those for the other yield components were observed. This low heritability can be explained by a stronger environmental influence on GY expression, which is a polygenic trait with predominance of dominance effects [7,8,57]. In turn, the highest heritability values for PE and yield components (100 GW, EL and ED) indicated a lower environmental influence on the expression of these traits. In particular, PE is an oligogenic trait, described by Dofing et al. (1991) [28], with predominance of components of genetic variance of additive effects [8]. The yield components, in spite of having a profile similar to GY, are less pronounced, which can be attributed to a weaker inbreeding depression effect on these traits than on GY [58,59].

Estimates of the coefficients of genetic variation (CVg) allow breeders to foresee the relative magnitude of changes that can be obtained by selection throughout a breeding program, since this parameter is directly proportional to genetic variance [60]. In general, the CVg of GY and PE were high, which indicates good chances of success in breeding programs that use these populations and select for these traits.

The ratio between CVg and CVe, known as the variation index, can be used to predict the success in the selection of superior genotypes, which will depend on its magnitude [32]. 

For 100 GW, CL and CD, the $I_{v}$ of the FSF and HSF progenies was greater than 1, indicating a more favorable situation of these characteristics to breeding; in this way, simple selection methods would be sufficient to obtain satisfactory gains.

For PE, the $I_{v}$ estimates of HSFs were generally between 0.57 and 1.12%. These estimates indicate the possibility of obtaining future gains through the advancement of generations. For the full-sib families, the values of $I_{v}$ (1.12 and 1.34%) indicated that PE is a trait for which gains are easily obtained, due to the lower environmental variation compared to genetic variation.

Values greater than 0.50% for GY regarding HSF_Pop2_ and FSF progenies indicate possibilities of future gains through selection. However, for HSF_Pop1_, although the frequency of favorable alleles for GY was high in the parents of the synthetic population, values lower than 0.50% were observed for the characteristic in both environments. The reason may be that the population had not been subjected to any selection cycle so far.

### 3.4. Expected Selection Gains

The key factors that can impair selection gains are selection intensity, the genetic properties of populations and environmental conditions [61]. In addition to these factors, the difficulty of selecting and obtaining superior cultivars in popcorn is increased by the existence of a negative correlation between GY and PE. In this context, in order to obtain simultaneous gains in both characteristics, selection indices are required. Among the classic selection indices, the one proposed by Mulamba and Mock (1978) [62] has been stressed in popcorn recurrent selection programs, as it provides the highest gains for the multitraits, favoring the selection of the best families [5,20,38]. Thus, selection gains are directly related to the difference in means between the selected groups and the original population.

Another important factor for the breeding program is the application of economic weights. The economic weights established by Cruz et al. (2012) [61] can be computed based on estimators ($SD_{g}$, $CV_{g}$, $I_{v}$, $h_{2}$), using values from proper experimental units or by means of arbitrary weights (AW) assigned by the breeder to establish the best gains for the breeding program.

The use of the estimator $CV_{g}\;$could be a good reference for the selection of superior families because it is closely related to genetic variance. However, $CV_{g}$ was not advantageous to provide considerable gains for GY, producing the lowest estimates (0.04 and 0.21%) and should therefore not be considered a superior estimator for selection.

The estimates of $I_{v}$ and $h_{2}$ as economic weights, despite the high gains for GY they provided, were not advantageous to allow considerable gains for PE, expressing the lowest estimates; in other words, these estimators should not be considered as alternatives to improve selection gains. 

The use of $SD_{g}$ estimates as economic weights predicted intermediate gains for GY (1.58 and 3.48%)while predicting some of the highest gains for PE (10.04 and 8.69). However, in this study, the proposed selection was directed towards an accumulation in greater simultaneous gains for the main economic traits in popcorn—GY and PE.

The arbitrary economic weights assigned by the breeder provided the highest gains for GY (8.72 and 8.55%) and PE (8.71 and 9.68%). By favoring higher simultaneous gains for GY and PE, the weights assigned by the breeder overcome the existence of a negative correlation between the two main economic characteristics in popcorn [8,11]. The intermediate gains obtained for EL (3.27 and 4.26%) will provide an increase in GY, since the greater the ear size, the greater the number of grains per productive unit. On the other hand, the decrease in 100 GW and ED will provide an increase in popcorn quality, which is related to a smaller grain size. Smaller grains are associated with a greater proportional popcorn volume [11,12]. Thus, the current pathway of popcorn breeding will balance increases in EL to obtain greater GY with decreases in 100 GW and ED to increase PE, in order to optimize gains for the traits of major interest.

In this respect, estimates of genetic gains show that interpopulation hybrids established at the end of each selection cycle may not only become genetic sources for the development of lines, but also have a yield, quality and competitive potential that can be marketed, serving as proof of the technical feasibility and economic viability of this methodology. It is noteworthy that the production costs of interpopulation hybrid seeds are lower than those of developing a single-cross hybrid; therefore, the first option is less costly for the breeder and, consequently, for the producer.

In popcorn, recurrent intrapopulation selection is being used to increase the frequencies of favorable alleles, predominantly for traits with high additive genetic effects [9,20,38]. However, this type of selection does not only favor gains in traits with a strong dominance effect, but also indicates the possibility of gains for traits in which dominance is not very strong, particularly for popping expansion. This makes interpopulation recurrent selection an advantageous strategy for popcorn breeding, as it allows the maintenance of genetic variability, with an increase in yield and a concomitant increase in the final product quality. In addition to allowing gains among populations with dominance by exploiting allelic complementation in hybrids, reciprocal recurrent selection increases the concentration of additive alleles and promotes gains within each breeding population, through selection and recombination of superior families [56].

## 4. Materials and Methods

### 4.1. Genetic Lines

**Figure 2:**
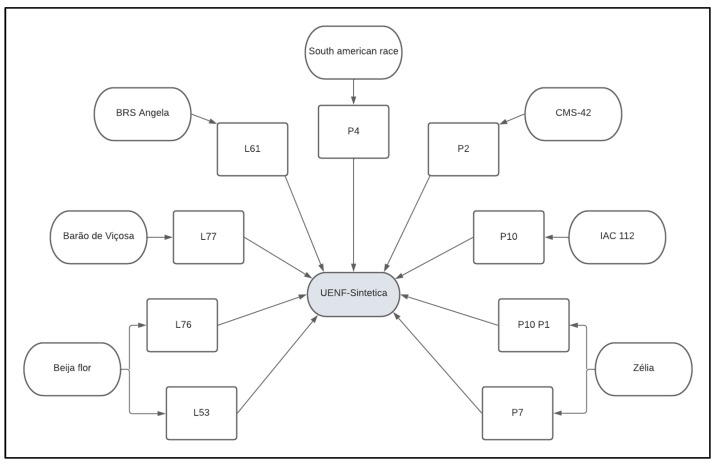
Genealogy chart of Pop_1_—UENF-synthetic of the popcorn breeding program of the State University of Northern Rio de Janeiro Darcy Ribeiro. Campos dos Goytacazes, Rio de Janeiro, Brazil.

**Figure 3:**
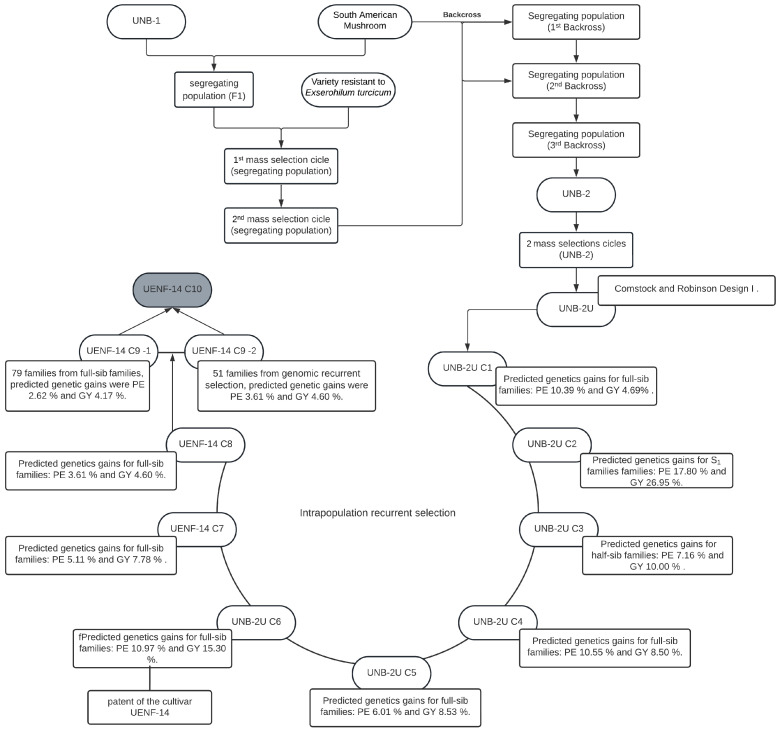
Genealogy chart of Pop2—cycle C10 of UENF-14 of the popcorn breeding program of the State University of Northern Rio de Janeiro Darcy Ribeiro. Campos dos Goytacazes, Rio de Janeiro, Brazil.

The composite populations for this study consisted of: (i) Population 1 (Pop_1_), resulting from random crosses of nine lines of the germplasm bank of the UENF popcorn breeding program. These lines had previously been evaluated and selected, based on the estimates of general combining ability for the two main characteristics of interest of the crop—grain yield and popping expansion—and on differences in their genealogical origins [12,63,64] (Figure 2). Pop. 1 was obtained through open pollination in an isolated field at the Experimental Station of PESAGRO-RIO, in Itaocara andRio de Janeiro, Northwest Fluminense region. Each line was sown in four 10.00 m long rows spaced 0.90 m between rows and 0.20 m between plants, using two plants per hole, making a stand of 100 plants per row. Once the synthetic population was obtained, it was subjected to recombination. For this process, the seeds were homogeneously mixed in bulk and subsequently sown in 30 rows following the same planting dimensions as in the previous process. (ii) Population 2 (Pop_2_) consisted of the intrapopulation recurrent selection C10 cycle from UNB-2U, which became designated UENF-14 from the sixth selection cycle. Cultivar UENF-14 started with the UNB-2U population, being an open-pollinated variety. Before arriving at UENF, this variety was made up of an indigenous population donated to the Universidade de Brasília by the Universidade de São Paulo/EALQ, which was named UNB-1. Subsequently, UNB-1 was brought to UENF by Professor Joachim Friedrich Wilhelm Von Bülow in 1993 and crossed with the popcorn variety SAM (South American Mushroom). This first filial generation was then crossed with a popcorn variety resistant to *Exserohilum turcicum*. After two mass selection cycles, three backcrosses were made with the SAM variety, thus originating the UNB-2 population. This population, after two rounds of mass selection, gave rise to the UNB-2U population. Nine cycles of recurrent selection were then carried out in order to obtain gains for the main characteristics of economic importance of the crop—expansion capacity and grain yield [5,11,21,30,36,37,38,40,65] (Figure 3). 

### 4.2. Obtaining Intrapopulation and Interpopulation Families

The methodology described here is the same as that proposed by Hallauer et al. (2010) [25]; therefore, to evaluate the per se performance of the populations, 200 half-sib families were obtained, 100 from Pop1 and 100 from Pop2. For the obtain half-sib families, 30 rows of the seed bulk of each population were sown, with dimensions of 10.00 m in length, 0.90 m between rows and 0.20 m between plants, leaving two plants per pit. Planting was carried out in temporally and spatially isolated fields at the PESAGRO-RIO Experimental Station in Itaocara, Rio de Janeiro. After the harvest, 100 families from each population were selected.

The full-sib families and S1 progenies were obtained by planting the two populations at Colégio Estadual Agrícola Antônio Sarlo, in Campos dos Goytacazes. The populations were sown in alternate rows measuring 6.00 m in length, with 1.00 m spacing between rows and 0.40 m spacing between plants. The rows and the plants of each row were numbered to facilitate identification when crossing. For each pair of plants, two reciprocal crosses (interpopulation hybrid) and two self-fertilizations (one in each population) were performed, thus constituting 100 full-sib families and 200 S1 progenies. The S1 seeds were stored in a cold room and the full-sib families were used in competition trials.

Pollinations were carried out manually, adopting the following procedure: the ear were covered before releasing the style–stigma with plastic bags suitable for this purpose. Simultaneously, the tassels were covered so that unwanted pollen contamination did not occur. This procedure is extremely necessary, as the pollen loses its viability eight hours after the beginning of its release; therefore, any viable pollen found in the paper bag the day after grooming could only have come from the covered tassel. Crosses occurred in prolific plants, selected within each pair of rows, so that the first (uppermost) ear resulted in the hybrid from which full-sibs were derived and the second ear resulted from selfing, to form the remaining seeds. The plants in each row were numbered for better control in identifying crosses.

### 4.3. Evaluation of Families and Experimental Design

The evaluation tests were carried out in the growing season of 2020/2021 in two environments: (i) Colégio Estadual Agrícola Antônio Sarlo in the district of Campos dos Goytacazes, northern region of the State of Rio de Janeiro (lat. 21°45′ S, long. 41°20′ W; 11 m asl), with a mean annual precipitation of 1023 mm and potential evapotranspiration of 1601 mm per year, at a mean annual temperature of 23 °C; and (ii) Experimental Station PESAGRO-RIO in Itaocara, Rio de Janeiro, in the northwest of the State of Rio de Janeiro (lat. 21°39′ S, long. 42°04′ W; 60 m asl), with a mean annual precipitation of 1041 mm and a mean annual temperature of 22.5 °C. The total distance between the locations is 110 km.

The experimental units consisted of 3.00 m long rows, spaced 0.90 m apart, with 15 plants per row. The experiment was arranged in an 18 × 18 lattice design, with three replicates. Thus, a total of 324 treatments were evaluated, i.e., 200 half-sib families (100 of Pop_1_ and 100 of Pop_2_), 100 full-sib families and 24 controls including the 10 cycles of recurrent intrapopulation selection of UENF-14, six populations from the popcorn germplasm bank of the State University of Northern Rio de Janeiro Darcy Ribeiro (UENF) and eight single-cross hybrids registered by the Ministry of Agriculture, Livestock and Food Supply. Crop treatments, fertilization and phytosanitary control were applied as recommended for the crop [66].

### 4.4. Evaluated Characteristics

The following agronomic characteristics were evaluated: mean grain yield (GY), estimated as the weight of grains harvested from the entire plot area and converted to t ha^−1^; popping expansion (PE; in mL g^−1^), determined by popping 30 g of kernels in the microwave oven for 2 min, measuring the popcorn volume in a 2000 mL beaker and dividing the popped volume by 30 g; mean 100-grain weight (100 GW; in g), obtained by assessing the weight of 100 randomly collected grains and weighing on an analytical balance; mean ear length (CL; in cm), by measuring five randomly picked ears without husk per plot; and mean ear diameter (CD; in cm), measured with a digital caliper in the middle of five random ears per plot.

### 4.5. Statistical Analyses

Initially, combined variance analysis was performed to detect the occurrence of significant interactions between the environments. For this purpose, we considered the main genotype effects as random and environment effects as fixed, in order to capitalize on possible interactions between them. The following statistical model was adopted: $Y_{ijkl}=µ+G_{i}+E_{l}+{\left(b/r/E\right)}_{j\left(k\right)\left(l\right)}+{\left(r/E\right)}_{k\left(l\right)}+{\left(GE\right)}_{il}+e_{ijkl}$ where $Y_{ijkl}$ is the observed value of the *i*-th genotype in the *j*-th block within the *k*-th repetition in the *l*-th environment; µ is the overall experimental constant; $G_{i}$ is the random effect of the *i*-th genotype, which is normally independently distributed (NID) (0, *σ*^2^*_g_*); $E_{l}$ is the fixed effect of the *l*-th environment; $b/r/e_{j\left(k\right)\left(i\right)}$ is the effect of the *j*-th block within the *k*-th repetition and within the *l*-th environment, which is NID (0, *σ*^2^*_b_*); $R/E_{kl}$ is the effect of the *k*-th repetition within the *l*-th environment; $GE_{il}$ is the interaction effect of the i-th genotype and the *l*-th environment, which is NID (0, *σ*^2^*_ge_*); and $e_{ijkl}$ is the mean experimental error associated with the $Y_{ijkl}$ observation, which is NID (0, *σ*^2^).

When a significant genotype × environment (GE) interaction effect was detected, the type of interaction (simple or complex) was specified, according to the method proposed by Cruz and Castoldi (1991) [67].

Based on the significance of the GE interaction, individual variance for each environment was analyzed based on the statistical model: $Y_{ijk}=µ+G_{i}+r_{j}+b/r_{k\left(j\right)}+e_{ijk}$ where $Y_{ijk}$ is the observed value of the *i*-th treatment in the *k*-th block, within the *j*-th repetition; µ is the overall experimental constant; $G_{i}$ is the random effect of the i-th treatment, which is NID (0, *σ*^2^*_G_*); $r_{j}$ is the effect of the *j*-th repetition; $b_{k\left(j\right)}$ is the random effect of the *k*-th incomplete block, ranked within the *j*-th repetition, which is NID (0, *σ*^2^*_B_*); and $e_{ijk}$ is the mean experimental error associated with the *Y_ijk_*, observation, according to the NID distribution, with mean 0 and variance *σ*^2^.

To estimate the genetic and environmental parameters, the following estimators were used: (a) Genotypic variance: $\sigma_{g}^{2}=\left(GMS-RMS\right)/r$ where GMS = genotype mean square, RMS = residual mean square, and r = number of repetitions; (b) Additive variance between families within populations: $\sigma_{a\left(within\right)}^{2}=4\times \sigma_{g\left(HSF\right)}^{2}$ where $\sigma_{g\left(HSF\right)}^{2}$ = genotypic variance between half-sib families; (c) Genetic variation coefficient: $CV_{g}\left(\%\right)=\left(100\sqrt{{\hat{\sigma }}_{g}^{2}}/\bar{X}\right)$ where ${\hat{\sigma }}_{g}^{2}$ = genotypic variance within families and $\bar{X}$ = overall mean of the trait within the family; (d) Heritability: $h^{2}=\frac{\sigma_{g}^{2}}{\sigma_{f}^{2}}$ where $\sigma_{g}^{2}$ = genotypic variance within families and $\sigma_{f}^{2}$ = phenotypic variance within families; (e) Index of variation:$\;I_{v}\;\left(\%\right)=100\left(\frac{CV_{g}}{CV_{e}}\right)$ where $CV_{g}\;$= genetic variation coefficient and $CV_{e}$ = experimental variation coefficient; and (f) Heterosis based on family means: $H\;\left(\%\right)=100\left(\frac{{\bar{X}}_{FSF}-{\bar{X}}_{HSF}}{{\bar{X}}_{HSF}}\right)$ where $\bar{X}F_{FSF}$ = mean of full-sib families and ${\bar{X}}_{HSF}$ = mean of half-sib families from the two populations.

### 4.6. Selection Strategy

Selection gains were estimated and superior full-sib families were selected by assigning different economic weights, at a selection pressure of 30% (30 families). To this end, covariances were estimated for all characteristics in both environments simultaneously, using the methodology of the sum-of-ranks index [67].

This sum-of-ranks index [62] consists of ranking the genotypes by assigning higher absolute values to those with the best performance, considering each evaluated characteristic. Subsequently, the sum of ranks was computed for each genotype and each studied characteristic, resulting in the selection index, calculated as:$\;I=r_{1}+r_{2}+\ldots +r_{n}$ where I is the index value for a given individual or family; $r_{j}$ is the classification (or rank) of an individual in relation to the *j*-th characteristic; and *n* is the number of characteristics considered in the index.

Using this method, different weights can be assigned to order the ranks of characteristics, as specified by the breeder. In this way, the economic weights were given by: $I=p_{1}r_{1}+p_{1}r_{2}+\ldots +p_{n}r_{n}$ where $p_{n}$ is the economic weight attributed to the *j*-th characteristic and $r_{j}$ is the classification (or rank) of an individual in relation to the *j*-th characteristic.

**Figure 4:**
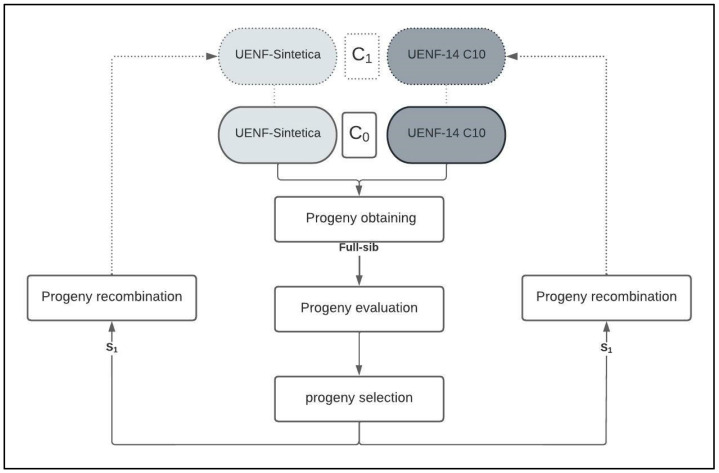
Flow chart of interpopulation recurrent selection in the popcorn breeding program at the State University of Northern Rio de Janeiro Darcy Ribeiro. Campos dos Goytacazes, Rio de Janeiro, Brazil.

The use of indices was based on the application of weights, where the assigned values were established by estimating genetic parameters and arbitrary values, namely the genotypic standard deviation (*SD_g_*), genetic variation coefficient (*CV_g_*), variation index (*I*_v_), heritability (*h*^2^) and economic weights assigned by the breeder (AW). In this context, after several arbitrary attempts, greater weights were applied to the most relevant characteristics for the crop under study: 10 for GY and PE and 1 for 100 GW, CL and CD. For all statistical genetic analyses, the resources of the program GENES [67] were used. After selection, the selected families were recombined, using the remaining seeds (S_1_) (Figure 4).

## 5. Conclusions

In popcorn breeding, exploiting heterosis is a promising strategy, mainly with a view to increasing grain yield (GY) and its components.

The first cycle of recurrent selection among popcorn populations in the framework of the UENF breeding program produced seven hybrids in the environment of Itaocara and ten in Campos. The hybrid GY exceeded 3 t ha^−1^ and PE was higher than 30 mL g^−1^, indicating genetic potential for breeding new varieties.

The Mulamba and Mock index was efficient in predicting crosses for satisfactory genetic gains for grain yield and popping expansion, favoring the continuity of the popcorn breeding program. The economic weights assigned arbitrarily by the breeder provided the highest genetic gains for GY and PE in the first cycle of interpopulation recurrent selection.

Two cycles of intrapopulation recurrent selection in the UENF-synthetic population for the subsequent use in recombination with population UENF-14 would favor more marked gains in popping expansion in FSFs, which are also associated with relevant gains in GY.

## Figures and Tables

**Table 1:** Summary of combined analysis of variance, experimental variation coefficients (CVe %), overall mean and the type of environmental interaction for six traits evaluated in popcorn populations in the first cycle of interpopulation recurrent selection in popcorn grown in Campos dos Goytacazes and Itaocara.

	Mean Squares										
	DF	GY		PE		100 GW		EL		ED	
Genotype (g)	323	1.34	**	90.28	**	5.68	**	5.75	**	15.63	**
Group	3	34.39	**	3548.98	**	29.66	**	1.39	*	473.47	**
FSF	99	1.17	**	72.94	**	4.54	**	7.60	**	12.70	**
HSF_Pop1_	99	0.59	**	39.21	**	5.08	**	4.25	**	13.34	**
HSF_Pop2_	99	1.27	**	23.63	**	5.37	**	4.39	**	7.53	**
Control	23	1.22	**	220.55	**	11.36	**	10.65	**	13.19	**
Environment	1	3.92	**	126.13	**	1787.39	**	0.01	**	433.53	**
G × E	323	0.40	**	12.27	**	3.02	**	2.83	**	4.08	**
Group × E	3	3.14	**	87.00	**	7.01	**	0.58	ns	10.50	**
FSF × E	99	0.43	**	11.44	**	2.93	**	2.92	**	3.73	**
HSF_Pop1_ × E	99	0.37	**	8.84	ns	3.11	**	2.64	**	4.34	**
HSF_Pop2_ × E	99	0.33	*	13.89	**	3.18	**	2.95	**	4.31	**
Control × E	23	0.28	ns	13.93	**	1.74	**	2.96	**	2.59	**
Residue	1190	0.36		7.31		0.75		0.51		1.60	
CVe (%)		24.03		9.62		6.21		4.43		3.31	
Mean		2.30		28.09		13.95		16.17		31.13	
Simple Interact. (%)		36.58		54.76		0.14		16.79		38.80	
Complex Interact. (%)		63.42		45.24		99.86		83.21		61.20	

DF = degrees of freedom; GY = mean grain yield (t ha^−1^); PE = popping expansion (mL g^−1^); 100 GW = mean 100-grain weight (g); EL = mean ear length (cm); ED = mean ear diameter (mm); FSF = full-sib families; HSF_Pop1_ = half-sib family from population UENF-synthetic; HSF_Pop2_ = half-sib family from population UENF-14; CVe (%) = estimates of experimental variation coefficients. Simple Interact. = simple interaction (%); Complex Interact. = complex interaction (%); ** and * indicate a significant difference at the level of 1%, and 5% by the F test, respectively; ns: absence of significant difference by the F test at 5% probability.

**Table 2:** Summary of individual analysis of variance of six agronomic traits of popcorn in interpopulation recurrent selection in popcorn grown in Campos dos Goytacazes and Itaocara, Rio de Janeiro, Brazil.

		Mean Squares										
		DF	GY		PE		100 GW		EL		ED	
Itaocara	Repetitions	2	2.88		26.92		1.58		1.39		0.65	
	Block/repet. (aj)	51	0.73		7.76		1.01		0.61		1.21	
	Treat. (adjust.)	323	1.03	**	59.33	**	4.37	**	3.83	**	11.59	**
	Group	3	26.37	**	2353.82	**	23.48	**	1.35	*	310.85	**
	FSF	99	0.94	**	47.15	**	3.9	**	4.68	**	8.7	**
	HSF_Pop1_	99	0.55	**	27.54	**	4.42	**	3.24	**	11.14	**
	HSF_Pop2_	99	0.86	**	21.5	**	3.81	**	3.22	**	6.85	**
	Control	23	0.86	**	112.16	**	6.1	**	5.59	**	7.32	**
	Error	595	0.36		7.08		0.82		0.51		1.04	
	CVe		26.76		9.56		6.09		4.41		3.23	
	Mean		2.25		27.84		14.91		16.18		31.60	
Campos dos Goytacazes	Repetitions	2			4.07		2.97		1.1		0.73	
	Block/repet. (aj)	51	0.49		10.33		1.07		0.57		0.99	
	Treat. (adjust.)	323	0.71	**	43.23	**	4.33	**	4.75	**	8.11	**
	Group	3	11.15	**	1282.15	**	13.19	**	0.62	ns	173.11	**
	FSF	99	0.66	**	37.23	**	3.57	**	5.84	**	7.73	**
	HSF_Pop1_	99	0.41	**	20.5	**	3.78	**	3.64	**	6.53	**
	HSF_Pop2_	99	0.75	**	16.03	**	4.74	**	4.13	**	4.99	**
	Control	23	0.64	**	122.32	**	7	**	8.02	**	8.45	**
	Error	595	0.25		7.54		0.68		0.52		1.09	
	CVe (%)		21.23		9.69		6.33		4.45		3.4	
	Mean		2.34		28.35		12.99		16.17		30.66	

DF = degrees of freedom; GY = mean grain yield (t ha^−1^); PE = popping expansion (mL g^−1^); 100 GW = mean 100-grain weight (g); EL = mean ear length (cm); ED = mean ear diameter (mm); FSF = full-sib family; HSF_Pop1_ = half-sib family from population UENF-synthetic; HSF_Pop2_ = half-sib families from population UENF-14; CVe (%) = estimates of experimental variation coefficients; ** and * indicate a significant difference at the level of 1%, and 5% by the F test, respectively; ns: absence of significant difference by the F test at 5% probability.

**Table 3:** Estimates of variance components and genetic parameters for six agronomic traits of half-sib and full-sib families from the popcorn populations UENF-synthetic and UENF-14 grown in Campos dos Goytacazes and Itaocara, Rio de Janeiro, Brazil.

	Itaocara			Campos dos Goytacazes		
	FSF	HSF_Pop1_	HSF_Pop2_	FSF	HSF_Pop1_	HSF_Pop2_
Mean Grain Yield (GY)						
$\sigma_{g}^{2}$	0.19	0.06	0.16	0.14	0.05	0.17
$h^{2}$ (%)	61.28	34.26	57.45	62.73	39.35	66.75
$CV_{g}$ (%)	16.34	12.21	19.67	14.36	9.96	19.13
$I_{v}$ (%)	0.61	0.46	0.74	0.68	0.47	0.90
$\sigma_{a\;\left(HSF\right)}^{2}$		0.25	0.66		0.21	0.66
Popping Expansion (PE)						
$\sigma_{g}^{2}$	13.35	6.82	4.80	9.90	4.32	2.83
$h^{2}$ (%)	84.98	74.28	67.05	79.75	63.22	52.96
$CV_{g}$ (%)	12.81	10.73	7.06	10.88	8.04	5.48
$I_{v}$ (%)	1.34	1.12	0.74	1.12	0.83	0.57
$\sigma_{a\;\left(HSF\right)}^{2}$		27.28	19.22		17.28	11.32
Mean 100-grain Weight (100 GW)						
$\sigma_{g}^{2}$	1.03	1.20	0.99	0.96	1.03	1.36
$h^{2}$ (%)	78.89	81.35	78.35	81.05	82.10	85.75
$CV_{g}$ (%)	6.70	7.26	6.87	7.39	7.87	9.08
$I_{v}$ (%)	1.10	1.19	1.13	1.17	1.24	1.43
$\sigma_{a\;\left(HSF\right)}^{2}$		4.79	3.98		4.13	5.42
Mean Ear Length (EL)						
$\sigma_{g}^{2}$	1.39	0.91	0.90	1.78	1.04	1.20
$h^{2}$ (%)	89.15	84.35	84.23	91.13	85.77	87.43
$CV_{g}$ (%)	7.29	5.88	5.89	8.26	6.30	6.76
$I_{v}$ (%)	1.66	1.33	1.34	1.86	1.42	1.52
$\sigma_{a\;\left(HSF\right)}^{2}$		3.65	3.62		4.17	4.81
Mean Ear Diameter (ED)						
$\sigma_{g}^{2}$	2.55	3.37	1.94	2.21	1.81	1.30
$h^{2}$ (%)	88.06	90.68	84.84	85.91	83.31	78.18
$CV_{g}$ (%)	5.06	5.58	4.58	4.86	4.26	3.84
$I_{v}$ (%)	1.57	1.73	1.42	1.43	1.25	1.13
$\sigma_{a\;\left(HSFI\right)}^{2}$		13.47	7.75		7.25	5.20

GY = mean grain yield (in t ha^−1^); PE = popping expansion (in mL g^−1^); 100 GW = mean 100-grain weight (in g); EL = mean ear length (in cm); ED = mean ear diameter (in mm); FSF = full-sib family; HSF_Pop1_ = half-sib family from the UENF-synthetic population; HSF_Pop2_ = half-sib family from population UENF-14; $\sigma_{g}^{2}$ = genetic variance; $h^{2}$ = heritability; $CV_{g}$ = genetic variation coefficient; $I_{v}$ = variation index, and $\sigma_{a\;\left(HSF\right)}^{2}$2 = genotypic variance within half-sib families.

**Table 4:** Predicted percentage gains of full-sib families from the UENF-synthetic (Pop1) and UENF-14 (Pop2) popcorn populations, estimated using the Mulamba and Mock selection index, assigning different economic weights, at a selection pressure of 30% (30 families) for six agronomic traits in plants grown in Campos dos Goytacazes and Itaocara, Rio de Janeiro, Brazil.

	Characteristics	Estimated Selection Gains (%)				
		*SD_g_*	*CV_g_*	*I_v_*	*h* ^2^	*AW*
Itaocara	GY	1.58	0.04	4.38	4.50	8.72
	PE	10.04	2.01	0.46	−0.98	8.71
	100 GW	−1.24	−0.06	2.26	3.01	−0.96
	EL	1.97	0.32	5.30	4.93	3.27
	ED	−0.94	−0.14	2.35	2.53	−0.39
		3.48	0.21	5.14	6.26	8.55
Campos dos Goytacazes	GY	8.69	1.79	1.65	1.32	9.68
	PE	0.36	0.32	2.87	2.70	−0.57
	100 GW	4.19	0.88	5.04	5.09	4.26
	EL	−0.56	−0.03	2.21	2.30	−1.13
	ED					

GY = mean grain yield (t ha^−1^); PE = popping expansion (mL g^−1^); 100 GW = mean 100-grain weight (g); EL = mean ear length (cm); ED = mean ear diameter (mm); $SD_{g}$ = genotypic standard deviation; $CV_{g}$ = genetic variation coefficient; $h^{2}$ = heritability; $I_{V}$ = variation index; and AW = economic weights assigned by the breeder (10, 10, 1, 1 and 1).

## Data Availability

Not applicable.

## Supplementary Materials

The following supporting information can be downloaded at: https://www.mdpi.com/article/10.3390/plants12051056/s1, Figure S1. Mean distribution of predicted gains of selected superior full-sib families. GY = mean grain yield; PE = popping expansion; 100 GW = mean 100-grain weight; EL = mean ear length; ED = mean ear diameter; and FSF = full-sib family. Figure S2. Distribution of selected superior full-sib families and means of parent populations. GY = mean grain yield; PE = popping expansion; UENF-14 = Population UENF-14; and Synth = UENF-synthetic.
