# Supplementary material for: Recurrent Interpopulation Selection in Popcorn: From Heterosis to Genetic Gains
Source: Plants (Basel). 2023 Feb 27;12(5):1056. doi: 10.3390/plants12051056 (PMC10005362; doi:10.3390/plants12051056)
Supplement: Supplementary file 1 [file plants-12-01056-s001.zip › plants-2199885-supplementary.pdf]

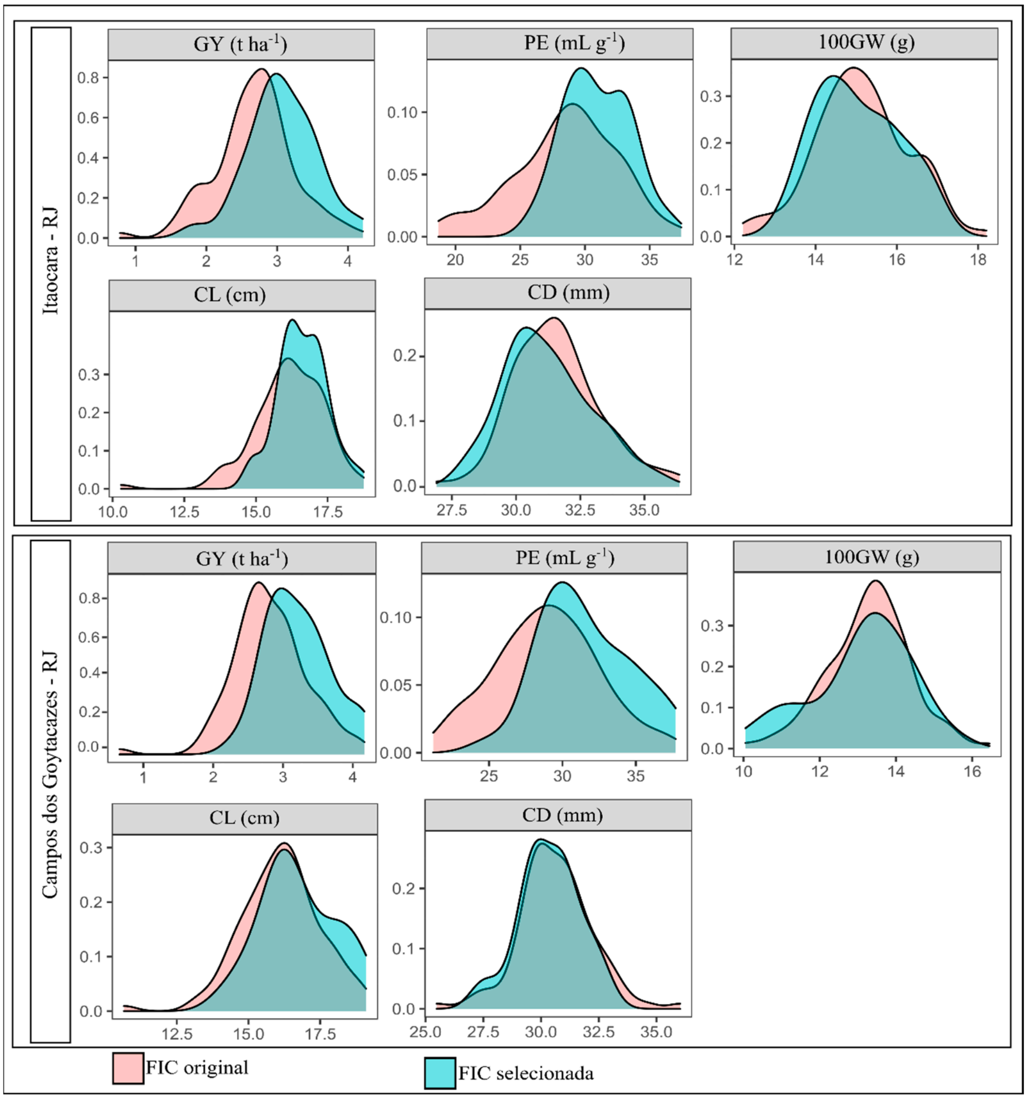

Figure S1. Mean Distribution of Predicted Gains of Selected Superior Full-Sib Families. GY = mean grain yield; PE = popping expansion; 100GW = mean 100-grain weight; EL = mean ear length; ED = mean ear diameter; and FSF = full-sib family.

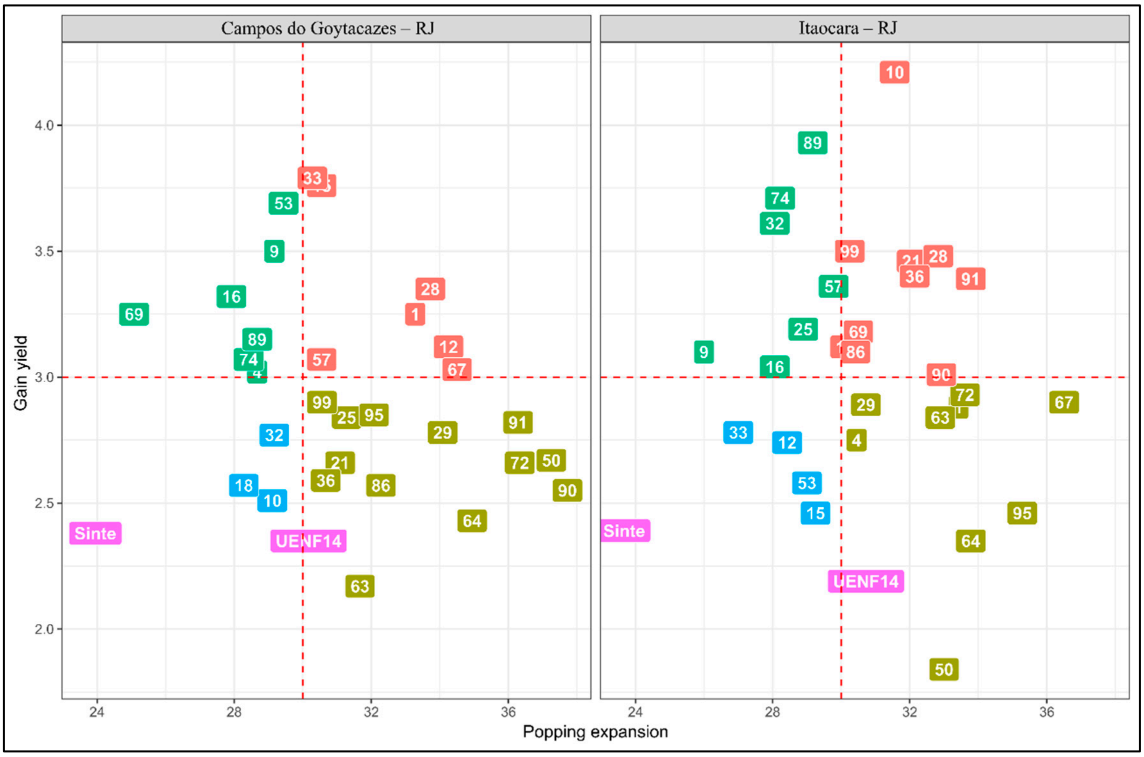

Figure S2. Distribution of selected superior full-sib families and means of parent populations. GY = mean grain yield; PE = popping expansion; UENF-14 = Population UENF-14; and Synth = UENF-synthetic.
